# Persistent Orthostatic Hypotension in a Patient With Acromegaly: Resolution With Transsphenoidal Hypophysectomy

**DOI:** 10.7759/cureus.24436

**Published:** 2022-04-24

**Authors:** Kassy E Kneen, Han G Ngo, Bipin Ghimire, Ajaz A Banka

**Affiliations:** 1 Internal Medicine, Oakland University William Beaumont School of Medicine, Royal Oak, USA; 2 Internal Medicine, Beaumont Hospital, Royal Oak, USA

**Keywords:** adrenal insufficiency, trans-sphenoidal resection, pituitary and diabetes, acromegaly and surgery, neurogenic orthostatic hypotension

## Abstract

Acromegaly is a rare condition characterized by excessive secretion of growth hormone from a pituitary tumor. It can affect multiple systems and can be fatal with cardiac dysfunction being the most common cause of death in these patients. Autonomic dysfunction is a less studied subject in patients with acromegaly, and the exact pathophysiology is still unclear. Here we present a case of a patient with persistent orthostatic hypotension, who was found to have acromegaly and pituitary adenoma upon further evaluation. Her orthostatic symptoms failed to improve with conservative measures and medical management, but unexpectedly resolved after transsphenoidal hypophysectomy was performed.

## Introduction

Acromegaly is a rare condition that occurs in three to four cases per one million people [1]. These patients typically present with frontal bossing, increased hat size, ring size, and shoe size due to the effects of growth hormone (GH) hypersecretion [2]. Early diagnosis of this condition is crucial because any delay can lead to cardiac dysfunction, which is the most common cause of death in these patients [1].

The exact pathophysiology of autonomic dysfunction associated with acromegaly is unclear as there have been only a few cases reported [3-5]. A case report by Kotwal et al. discussed an acromegalic patient with orthostatic hypotension who was able to be adequately treated with conservative measures [6]. Here we present a patient with acromegaly and persistent orthostatic hypotension whose symptoms failed to improve with conservative measures and medical management, but improved immediately after transsphenoidal hypophysectomy.

## Case presentation

A 41-year-old female with a medical history of pituitary adenoma, type 2 diabetes mellitus with peripheral neuropathy and gastroparesis, and neurogenic bladder with chronic indwelling Foley catheter initially presented to the emergency department with an episode of presyncope and a few days of pelvic discomfort. She also complained of lightheadedness, nausea, and worsening blurry vision every time she stood up from her bed. She had a history of blurred vision with floaters secondary to diabetic proliferative retinopathy, which was stable prior to presentation. Her systolic blood pressure was in the range of 110-120 mm Hg while supine, and decreased to 65-75 mm Hg upon standing. Her temperature, pulse, and oxygen saturation (SPO2) were normal. Initial investigations revealed normal sodium, potassium, bicarbonate, creatinine levels and anion gap. A 12-lead EKG showed sinus tachycardia. Urinalysis was suggestive of urinary tract infection (with high WBC, leukocyte esterase). Physical exam showed moist mucous membranes and no other signs of dehydration, suprapubic tenderness, and costovertebral angle tenderness. Neurological exam showed patient was alert and oriented to person, place, and time, pupils were equal, round, and reactive to light and accommodation, extraocular movements were intact, and there were no focal neurologic deficits. On initial presentation, signs and symptoms of acromegaly were not fully assessed.

The patient has a significant history of type 2 diabetes mellitus and its associated complications. Her first diagnosis of type 2 diabetes was established seven years ago. She was started on metformin at that time and was stopped after one year of therapy. She was off medical management of her diabetes for the next six years. Her most recent admission was four months prior to her presentation with severe diabetic ketoacidosis. During that admission, her hemoglobin A1c was 18.7%. On discharge, she was started on insulin and has been compliant since then. Her most recent hemoglobin A1c prior to her current admission was improved to 9%. She has been diagnosed with complications of diabetic retinopathy, neuropathy, and neurogenic bladder requiring chronic foley placement complicated by recurrent urinary tract infections. Additionally, she has history of premature menopause at the age of 37 and pituitary microadenoma. At the time of the imaging diagnosis of her pituitary microadenoma five months before her admission, her pituitary function was evaluated. At that time, her follicle-stimulating hormone (FSH), luteinizing hormone (LH), thyroid-stimulating hormone (TSH), prolactin, and insulin-like growth factor 1 (IGF-1) levels were all within normal limits. Therefore, her pituitary function was intact. Her home medications included: gabapentin 300 mg three times daily, NovoLog® 16 units with sliding scale three times daily with meals, insulin glargine 36 units once daily, Reglan® 5 mg three times daily as needed, and Bactrim DS twice daily.

After initial evaluation, the patient was started on antibiotics. Initially, it was presumed that her orthostatic hypotension and symptoms were due to hypovolemia in the setting of urinary tract infection (UTI) even though she did not have other signs or symptoms suggesting dehydration (normal mucus membranes, normal urine output). She received an initial bolus of isotonic fluids and maintenance fluids. She also started using compression stockings. With no resolution of symptoms, midodrine was added. She was started on 5 mg three times daily and was then gradually increased to 10 mg three times daily, but her symptoms did not improve. Given her history of pituitary adenoma, she was then evaluated for secondary adrenal insufficiency, which revealed borderline abnormal cosyntropin stimulation test with low baseline cortisol level 2.4 mcg/dl (normal 2.9 - 19.4), and cortisol level 30 minutes after stimulation being 16.3 mcg/dl (normal >18 mcg/dl). She was then started on hydrocortisone due to concern for adrenal insufficiency even though the test was only borderline positive. Her initial dose was 10 mg in the morning and 5 mg in the afternoon. This was increased to 10 mg twice daily and then escalated to 10 mg in the morning, 10 mg in the afternoon, and 5 mg in the evening. Despite these dosage increases, her symptoms did not improve after 13 days of hydrocortisone therapy. Since her symptoms did not improve with concurrent fluid resuscitation (total of 6L isotonic fluids in the admission), fludrocortisone was not started. 

**Figure 1:**
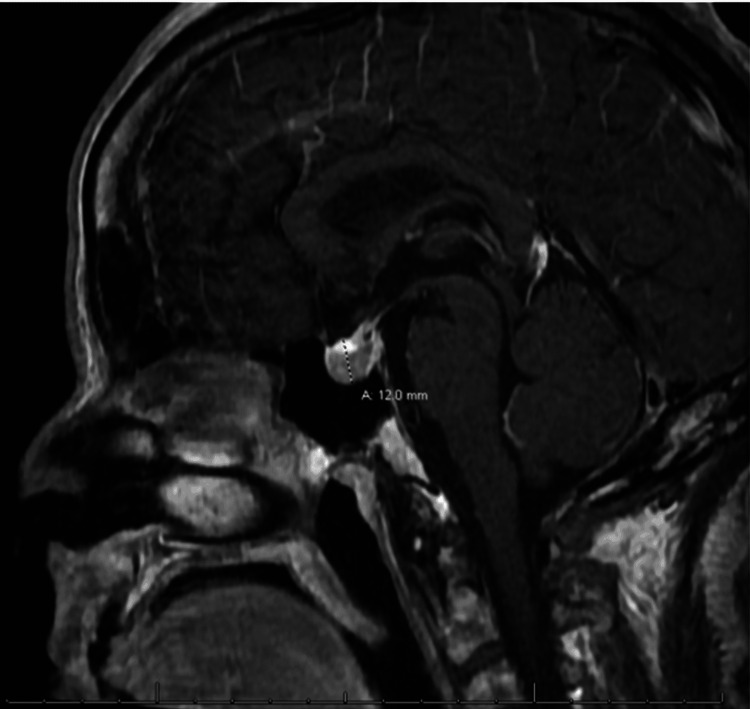
MRI brain sagittal view of 12 mm pituitary adenoma. Post-operative MRI showed complete resolution of pituitary adenoma.

After the borderline cosyntropin stimulation results, endocrinology was consulted on day six of admission. Upon further review of systems, she denied visual field defects or any new changes to her vision during this admission; she did not have peripheral edema, increased sweating or other complaints pertaining to acromegaly. However, the patient did complain of her ring being tighter and her shoe size had increased in the past two to three months. With her history of pituitary adenoma and these new complaints, further workup was done. MRI brain demonstrated persistently enlarged pituitary gland (dimension 12mm, Figure 1), which appeared to have increased in size compared to five months prior (5 mm x 8 mm). Further workup showed significantly elevated IGF-1 at 993 ng/mL on day six of admission and repeat level was 930 ng/mL on day eight of admission (normal 58-219 ng/mL). Additionally, her hemoglobin A1c was shown to be 8.3%. 

Neurosurgery was consulted, and the initial plan was to perform trans-sphenoidal pituitary adenoma resection as outpatient after discharge. However, since the patient did not feel safe returning home given her unresolving symptoms, this surgery was performed during the same admission. Since ophthalmology team usually performed complete and formal retinal and visual field exam as outpatient, it could not be completed prior to the surgery while she was still admitted. She received two doses of IV hydrocortisone 100 mg prior to the surgery, and continued to receive maintenance fluids during the surgery. On post-operative day one, the patient was re-evaluated for orthostatic hypotension. Surprisingly, she did not have any symptoms suggestive of orthostatic hypotension, and her systolic blood pressures were within normal range (110-130 mm Hg) on both supine and standing. She continued to have normal blood pressure throughout the admission. She continued to require similar insulin dosages as the pre-operative period. She was also continued on hydrocortisone after surgery. Repeat AM cortisol post-operatively was normal; however, cosyntropin stimulation test was not repeated.

## Discussion

Acromegaly is a disorder that affects multiple organ systems in the body, including the cardiac and nervous systems. Autonomic dysfunction in acromegaly is an often-overlooked complication of the disease. In a cross-sectional study with matched controls, Oz et al. describe the presence of autonomic dysfunction in 18 patients with acromegaly using sympathetic skin response (SSR) as an indicator for sympathetic sudomotor activity and RR interval variation (RRIV) as an indicator of cardiovagal autonomic function [4]. Similarly, Dural et al. [3] and Resmini et al. [5] demonstrate evidence of sympathovagal imbalance in acromegalic patients; however, this study was limited to cardiac autonomic function evaluation, and patients did not have symptoms or signs of orthostatic hypotension. Kotwal et al. presented a case of autonomic dysfunction with orthostatic hypotension that was attributed solely to acromegaly [6], but, unlike our patient, improved with conservative measures such as lifestyle changes to improve orthostatic hypotension, compression socks, and exercises. With these limited studies, the exact cause for autonomic dysfunction in acromegalic patients is still unknown. 

The most likely cause of autonomic dysfunction in acromegaly is due to sympathovagal imbalance, but this autonomic dysfunction can also be explained by diabetes mellitus which is the most common cause of autonomic neuropathy in the developed world [7]. Excess growth hormone can result in elevated blood glucose and subsequently diabetes mellitus, a very frequent complication of acromegaly [8]. Thus, diabetes mellitus was included in our differential diagnosis for orthostatic hypotension for this patient. Studies have shown improvement in glucose metabolism and insulin sensitivity in diabetic patients with acromegaly after adenoma resection; however, the follow-up in those studies has only been a few months post-surgery [9-10].

Since the patient’s blood glucose remained similar to pre-operative levels and she continued to require a similar dosage (units) of insulin for glucose control to her pre-admission regimen, it is likely that diabetes may not be the only cause of her orthostatic hypotension. Adrenal insufficiency was initially considered a possible cause but it was deemed less likely later in the course as the patient only had a borderline cosyntropin stimulation test without other suggestive clinical or laboratory findings, and was unresponsive to 13 days of hydrocortisone and several intravenous fluid boluses. Since her symptoms improved immediately after transsphenoidal hypophysectomy, we believe that acromegaly was a significant underlying cause of this patient’s orthostatic hypotension.

Treatment of orthostatic hypotension can be done conservatively with lifestyle changes, hydration, and medications such as fludrocortisone or midodrine [11]. However, in cases of orthostatic hypotension secondary to acromegaly, we have found pituitary adenoma resection to be a potential definitive treatment.

## Conclusions

Acromegaly is a rare disorder, and patients can present with orthostatic hypotension. Further evaluation for diagnosis should be performed if any subtle signs or symptoms to suggest the disorder are present. Conservative measures like hydration and medications can be used, but symptoms can be refractory to these measures. Pituitary adenoma resection may be the definitive management in such patients, even if other traditional indications for surgery are not present.
